# Multilocus sequence typing and genomic characterization of the novel *Streptomyces* strain SCPE-10, that resists 2,4-D toxicity and has bioremediation potential

**DOI:** 10.1007/s42770-026-01875-2

**Published:** 2026-02-24

**Authors:** Nildo Alfredo Nhampossa, João Victor Castro de Almeida Araújo, Vicente Almeida Serafim da Silva, João Ricardo Vidal Amaral, Cinara Souza da Conceição, Carlos Alberto Xavier Gonçalves, Eamim Daidrê Squizani, Sheila da Silva, Marcia Soares Vidal, Yinglong Chen, Andrew Macrae, Rodrigo Pires do Nascimento

**Affiliations:** 1 Laboratorio de Ecologia e Processos Microbianos-LEPM 108, Escola de Química, Centro de Tecnologia, Universidade Federal do Rio de Janeiro, Av. Athos da Silveira Ramos, 149 - Cidade Universitária, Rio de Janeiro, RJ 21941-909 Brazil; 2 Programa de Pós-Graduação de Biotecnologia Vegetal e Bioprocessos da Universidade Federal do Rio de Janeiro, Av. Prof. Rodolpho Paulo Rocco, s/n-Prédio do CCS-Bloco K, 2° Andar-Sala 032, Rio de Janeiro, 21941-902 Brazil; 3 Laboratório de Biotecnologia Sustentável e Bioinformática Microbiana, Departamento de Microbiologia Geral, Instituto de Microbiologia, Universidade Federal do Rio de Janeiro, Cidade Universitária, Rio de Janeiro, 21941-902 Brazil; 4 Instituto SENAI de Inovação em Biossintéticos e Fibras, Cidade Universitária, Rio de Janeiro, 21941-857 Brazil; 5 Embrapa Agrobiologia, Rodovia BR 465, km 07, Seropédica, RJ 23891-000 Brazil; 6 School of Agriculture and Environmental Science, University of Western Australia, Perth, Australia

**Keywords:** *Streptomyces* SCPE-10, 2,4-D degradation, Aromatic compounds, Xenobiotic biodegradation, Bioremediation

## Abstract

The widespread use of aromatic herbicides such as 2,4-dichlorophenoxyacetic acid (2,4-D) has led to persistent environmental contamination, requiring efficient and sustainable biodegradation strategies. In this study, we isolated and characterized a novel actinobacterial strain, *Streptomyces* sp. SCPE-10, from contaminated coastal soil, capable of using 2,4-D as its sole carbon source. Phenotypic assays revealed robust growth on aromatic substrates, while whole-genome sequencing (Submission no. *SUB15461668*) followed by multilocus sequence analysis (16 S rRNA, recA, rpoB, atpD, gyrB, and trpB) revealed that SCPE-10 is closely related to *Streptomyces phaeoluteichromatogenes*. Functional genome annotation revealed a high abundance of genes involved in aromatic compound degradation, including cytochrome P450 monooxygenases, ring-cleaving dioxygenases, and dehalogenases. KEGG and antiSMASH analyses identified multiple metabolic pathways and 28 biosynthetic gene clusters (BGCs), including clusters for polyketides, nonribosomal peptides, terpenes, siderophores, and ectoine. Notably, SCPE-10 harbors key genes related to the degradation of benzoate, naphthalene, toluene, xylene, and 2,4-D, indicating broad-spectrum catabolic potential. These findings suggest that *Streptomyces* sp. SCPE-10 is a promising candidate for the bioremediation of herbicide-contaminated environments and the exploration of novel secondary metabolites.

## Introduction

The widespread contamination of ecosystems by aromatic compounds including, synthetic herbicides, industrial dyes, and polycyclic aromatic hydrocarbons (PAHs), represents a persistent environmental concern with direct implications for human and ecological health [1–3]. Biodegradation has emerged as a sustainable alternative for mitigating these pollutants, with members of the phylum Actinomycetota, particularly those from the genus *Streptomyces*, playing a central role due to their rich metabolic repertoire and ecological adaptability [4–6]. The elucidation of the molecular mechanisms underlying these catabolic abilities requires comprehensive genomic and functional analyses. Advanced bioinformatic tools such as antiSMASH have enabled the identification of biosynthetic gene clusters (BGCs), which often encode key enzymes involved in biodegradation pathways [7]. Additionally, databases like KEGG provide detailed insights into metabolic routes associated with the oxidation and cleavage of aromatic rings, involving enzymes such as monooxygenases, dehydrogenases, and dioxygenases [8–10]. In this study, we report on the complete genome sequencing and genome annotation of *Streptomyces* sp. strain SCPE-10 and perform a comprehensive genome mining analysis aimed at identifying genes and BGCs related to the degradation of aromatic compounds. Using a combined approach based on antiSMASH and KEGG pathway analysis, we explore the metabolic potential of SCPE-10, with particular focus on its capacity to degrade environmental pollutants of aromatic nature. This work highlights the ecological and biotechnological potential of SCPE-10 for future applications in environmental bioremediation.

## Materials and methods

### Microbial strain

*Streptomyces* sp. SCPE-10 was selected from a screening of approximately 710 actinobacteria strains from the culture collection of the Laboratory of Microbial Process Engineering at UFRJ (LEPM-EQ/UFRJ). All isolates were grown on ISP-2 at 28 °C and pH 7.0. for 10 days, and the fastest-growing strains were tested for tolerance to 250 ppm of 2,4-dichlorophenoxyacetic acid (2,4-D) under three conditions: (1) with 1.0% malt extract; (2) with 0.5% malt extract; and (3) 2,4-D as the sole carbon source. SCPE-10 showed robust growth in some conditions, including minimal medium, and was thus selected for further biodegradation and genomic analysis.

### Phenotypic characterization

Colony morphology of *Streptomyces* strain SCPE-10 was evaluated after growth on KB and ISP-4 (International *Streptomyces* Project medium), incubated at 28 ± 1 °C for up to 10 days. An overnight culture from Gause #1 medium was streaked and observed for features such as color, form, and transparency. All assays were performed in triplicate.

### Ability of *Streptomyces* SCPE 10 to degrade aromatic compounds

The ability of *Streptomyces* sp. SCPE-10 to degrade the herbicide 2,4-dichlorophenoxyacetic acid (2,4-D) was initially tested on solid media containing 250 ppm 2,4-D with (1) 1.0% or (2) 0.5% malt extract, and (3) 2,4-D as sole carbon source. SCPE-10 only grew in media supplemented with malt extract, suggesting co-metabolism is required. A spore suspension was prepared from ISP-2 plates, and spores were harvested, filtered, and stored in 35% glycerol (4.97 × 10^10^ CFU/mL). Submerged fermentation was performed in 250 mL medium (250 ppm 2,4-D + 1.0% or 0.5% malt extract), inoculated with 897 µL of the spore suspension, and incubated at 28 °C, 180 rpm, for 14 days. Samples were collected at defined intervals and stored for enzymatic and degradation analyses.

### Genome sequencing, library construction, and annotation

Genomic DNA was extracted using phenol–chloroform, and sequenced with PacBio HiFi. Assembly was done with Hifiasm and annotated via Prokka v1.14.6. Completeness was assessed with BUSCO (Benchmarking Universal Single-Copy Orthologs) against the Actinobacteria database. Identification was confirmed by 16 S rRNA analysis using EzBioCloud (https://kb.ezbiocloud.net/home/protocols/16s-identication*)* and multilocus sequence analysis (MLSA) with six housekeeping genes: 16 S rRNA (16 S ribosomal RNA), recA (recombinase A), rpoB (RNA polymerase subunit beta), atpD (ATP synthase subunit beta), gyrB (DNA gyrase subunit B), and trpB (tryptophan synthase subunit beta), aligned and phylogenetically analyzed in Geneious v2025.0.3. (https://www.geneious.com*).* Functional annotation was performed with antiSMASH v7.0 (https://antismash.secondarymetabolites.org/*)* (secondary metabolites), KEGG (https://www.genome.jp/kegg/pathway.html*)* (xenobiotic metabolism), and MobileOG-db (mobile elements).

### In silico identification of aromatic compound degradation genes

The functional annotation of the *Streptomyces* sp. SCPE-10 genome was carried out to identify genes involved in the degradation of aromatic xenobiotics. Genome annotation was performed using the KEGG Orthology-Based Annotation System (https://www.genome.jp/brite/ko00001*)* and the RAST server. Enzymatic functions related to the degradation of chlorinated aromatic compounds were investigated, with emphasis on genes associated with the catabolism of 2,4-dichlorophenoxyacetic acid (2,4-D). The search focused on coding sequences annotated as dioxygenases (including α-ketoglutarate-dependent enzymes), monooxygenases, hydrolases, and dehalogenases. Genes homologous to members of the *tfd* cluster were identified based on conserved domains and sequence similarity. The presence of catechol 1,2-dioxygenase and catechol 2,3-dioxygenase genes was used to infer the potential for both ortho- and meta-cleavage of aromatic rings. Secondary metabolite biosynthetic gene clusters (BGCs) were detected using antiSMASH v7.0, and their colocalization with xenobiotic degradation-related genes was assessed to explore possible functional synergies.

### Data availability

The *Streptomyces* sp. SCPE-10 genome has been deposited and is available in the National Center for Biotechnology Information (NCBI) under the Submission number *SUB15461668*.

## Results

### Phenotypic characterization and ability to degrade aromatic compounds

**Fig. 1:**
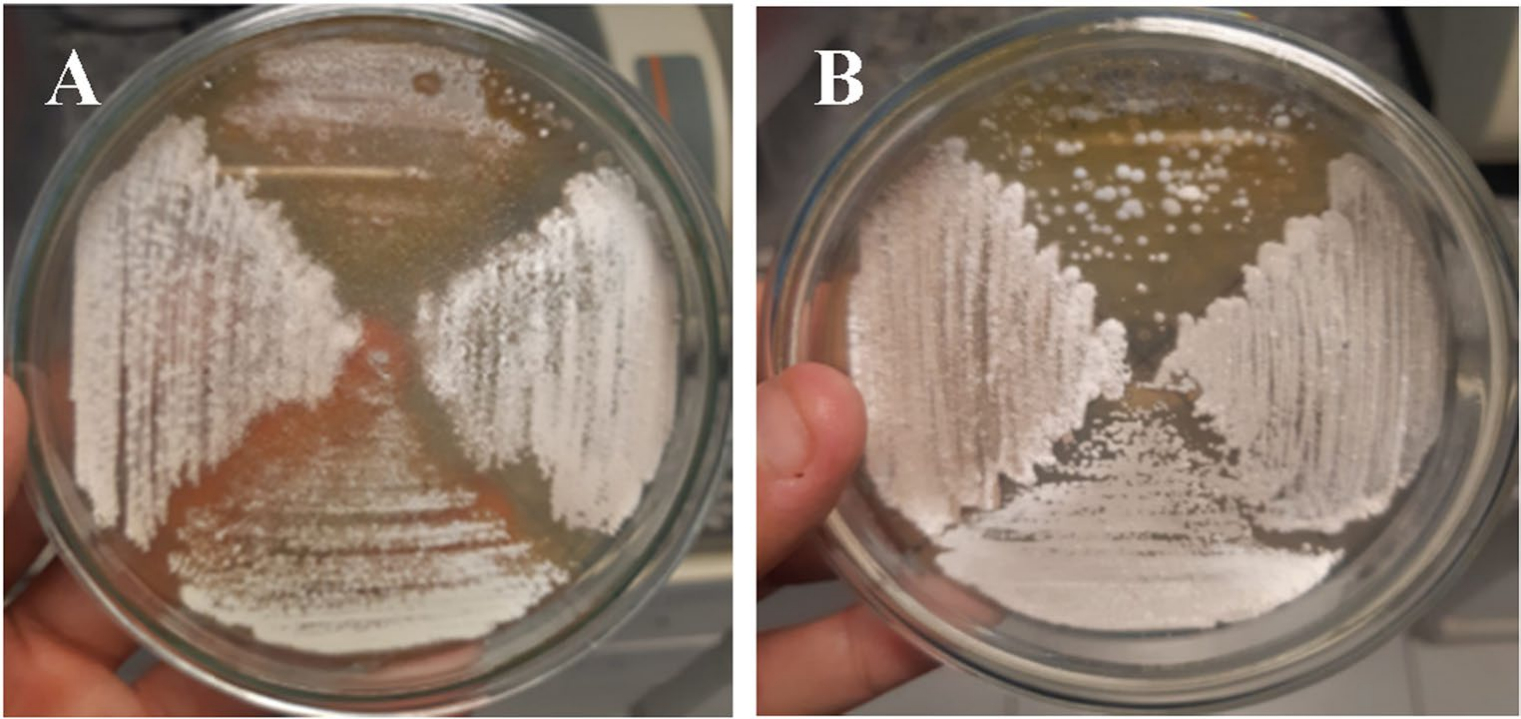
Colony morphology of *Streptomyces* sp. SCPE-10 on ISP-2 agar medium viewed from different angles. (**A**) Top view showing colony pigmentation and structure. (**B**) Close-up of aerial mycelium on colony surface showing typical growth after 10 days of incubation at 28 ± 1 °C

**Fig. 2:**
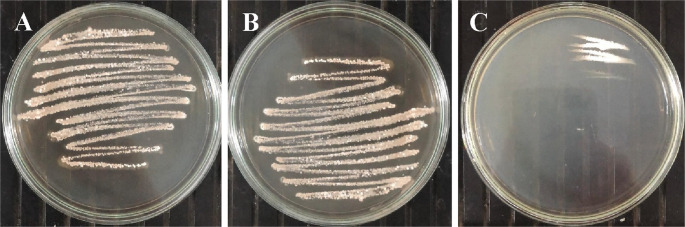
Growth of *Streptomyces* sp. SCPE-10 on ISP-2 agar supplemented with 2,4-D under varying nutrient conditions. (**A**) Medium 1: 2,4-D (250 ppm) + 1% malt extract. (**B**) Medium 2: 2,4-D (250 ppm) + 0.5% malt extract. (**C**) Medium 3: 2,4-D (250 ppm) without malt extract. Plates were incubated for up to 10 days at 28 ± 1 °C

**Fig. 3:**
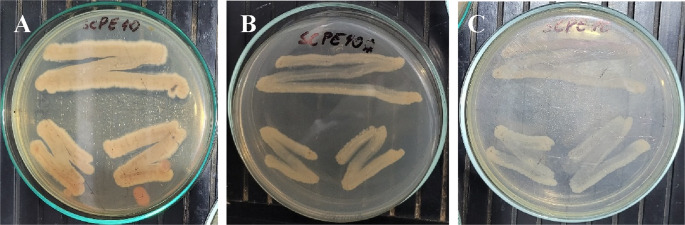
Comparative analysis of filamentous growth of *Streptomyces* sp. SCPE-10 on media with and without 2,4-D. (**A**) Mycelial density in Medium 1 indicates efficient growth. (**B**) Reduced mycelial development in Medium 2. (**C**) Repressed growth in Medium 3. Plates were incubated for up to 10 days at 28 ± 1 °C

After 10 days of incubation on ISP-2 medium at 28 ± 1 °C, *Streptomyces* sp. SCPE-10 formed buried, dry, and firm colonies with a white to beige pigmentation, typical of actinomycetes (Fig. 1A–B). The strain’s ability to metabolize aromatic compounds was assessed using ISP-2-based media supplemented with 250 ppm 2,4-dichlorophenoxyacetic acid (2,4-D) under three nutritional conditions. Robust growth was observed in Medium 1 (1.0% malt extract) and consistent growth in Medium 2 (0.5% malt extract), whereas no growth occurred in Medium 3, which contained only 2,4-D as the sole carbon source (Figs. 2A–C). This indicates the requirement of co-metabolism for 2,4-D degradation. Colony morphology remained filamentous and branched in Mediums 1 and 2, while no sporulation or expansion was noted under carbon-limited conditions (Fig. 3A–C). Among 11 screened isolates, only SCPE-10 and C2B grew consistently on 2,4-D media, with SCPE-10 selected for further genomic investigation due to superior performance.

### Genome sequencing analysis

**Fig. 4:**
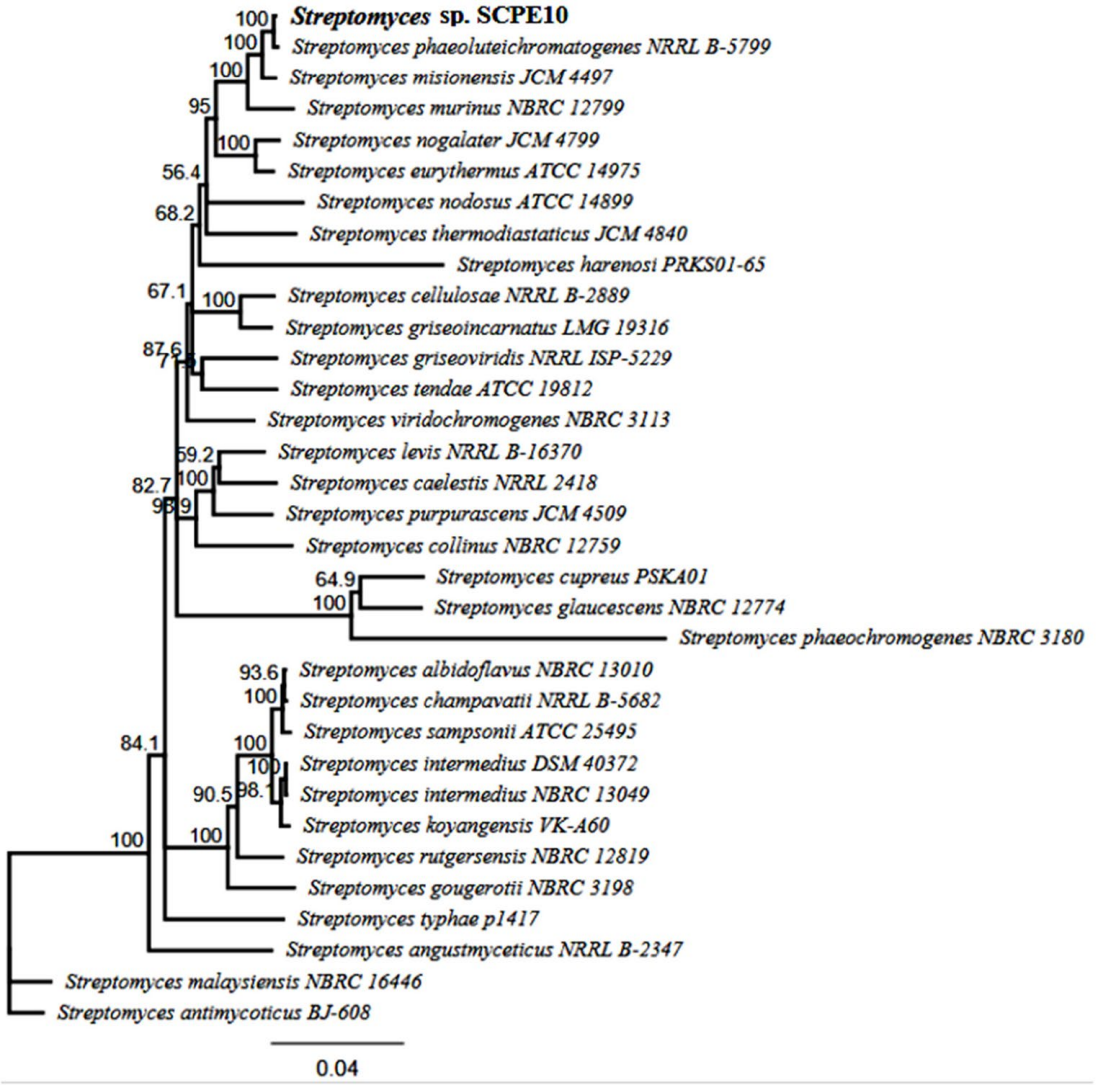
Maximum-likelihood phylogenomic tree based on 33 concatenated core genes, constructed using the Geneious tool, which automates the extraction and analysis of multilocus sequence typing (MLST) data from genomic sequences. The tree includes both query strains (QSs) and type strains (TSs), which serve as references. The bar indicates sequence divergence

The complete genome of *Streptomyces* sp. SCPE-10, sequenced via PacBio HiFi, consists of a linear chromosome with 8,012,345 bp and 72.3% G + C content, assembled into a single contig without plasmids. Prokka annotation predicted 7,222 genes, including 6,982 protein-coding sequences and 145 RNAs (90 tRNAs and 18 rRNAs) (Table 1). Taxonomic analysis based on 16 S rRNA via EzBioCloud revealed 99.86% similarity to *Streptomyces phaeoluteichromatogenes* (93.8% query coverage) (Table 2). MLSA using six housekeeping genes (16 S rRNA, recA, rpoB, atpD, gyrB, trpB) confirmed its close affiliation with *S. phaeoluteichromatogenes* (Fig. 4).

BUSCO analysis indicated 99.9% genome completeness. Annotation revealed genes related to xenobiotic degradation, including cytochrome P450 monooxygenases, ring-hydroxylating dioxygenases, and multicopper oxidases, as well as carbohydrate-active enzymes like chitinases and cellulases, suggesting ecological adaptability. AntiSMASH v7.0 identified 24 biosynthetic gene clusters (BGCs), including NRPS, PKS, siderophores, lanthipeptides, and terpenes, some similar to known BGCs (e.g., actinomycin D, geosmin), others potentially novel (Table 3). KEGG pathway analysis highlighted genes linked to the degradation of chlorinated aromatics, nitroaromatics, and PAHs, reinforcing the strain’s biotechnological potential (Table 4).

**Table 1:** Summary of genome assembly and annotation of *Streptomyces* sp. SCPE-10

Assembly	
Genome size (bp)	8,012,345
Number of contigs	1
GC content (%)	72.3
Genes (total)	7222
CDSs (total)	7113
CDSs (with protein)	6982
tRNAs	90
rRNAs	18
tmRNAs	1
Pseudogenes	Not detected
Sample ID	Strain SCPE 10
Assembly name	Streptomyces_SCPE 10
Organism	Streptomyces SCPE 10
Raw reads (fastq)	287 MB
Assembled genome (fasta)	7.7 MB
Taxonomic classification	Streptomycetales 99.9%

**Table 2:** Taxonomic identification of SCPE-10 was refined based on 16 S rRNA sequence analysis

#	Species	Genome group	Taxonomy	Iden.	Query	Ref.
				(%)	cov. (%)	cov. (%)
1	*Streptomyces phaeoluteichromatogenes*	-	Bacteria Actinobacteria Actinomycetia	99.86	93.80	100
			Streptomycetales Streptomycetaceae			
			*Streptomyces*			
2	*Streptomyces misionensis*	-	Bacteria Actinobacteria Actinomycetia	99.79	95.1	100
			Streptomycetales Streptomycetaceae			
			*Streptomyces*			
3	*Streptomyces malaysiense*	-	Bacteria Actinobacteria Actinomycetia	99.72	95.1	100
			Streptomycetales Streptomycetaceae			
			*Streptomyces*			
4	*Streptomyces cupreus*	-	Bacteria Actinobacteria Actinomycetia	99.17	95.1	100
			Streptomycetales Streptomycetaceae			
			*Streptomyces*			
5	*Streptomyces diastaticus*	-	Bacteria Actinobacteria Actinomycetia	99.03	95.0	100
			Streptomycetales Streptomycetaceae			
			*Streptomyces*			
6	*Streptomyces intermedius*	*Streptomyces albidoavus* group	Bacteria Actinobacteria Actinomycetia	99.03	95.0	100
			Streptomycetales Streptomycetaceae			
			*Streptomyces*			
7	*Streptomyces levis*	-	Bacteria Actinobacteria Actinomycetia	98.95	94.1	100
			Streptomycetales Streptomycetaceae			
			*Streptomyces*			
8	*Streptomyces glaucescens*	-	Bacteria Actinobacteria Actinomycetia	98.89	95.0	100
			Streptomycetales Streptomycetaceae			
			*Streptomyces*			
9	*Streptomyces collinus*	*Streptomyces iakyrus* group	Bacteria Actinobacteria Actinomycetia	98.89	94.4	100
			Streptomycetales Streptomycetaceae			
			*Streptomyces*			
10	*Streptomyces venetus*	-	Bacteria Actinobacteria Actinomycetia	98.83	95.1	100
			Streptomycetales Streptomycetaceae			
			*Streptomyces*			

**Table 3:** Secondary metabolic gene clusters predicted in the genome of *Streptomyces* sp. SCPE-10

Region	Type	Most similar known cluster		Similarity
1	terpene	herbimycin A	Polyketide	6%
2	redox-cofactor	lankacidin C	NRP + Polyketide	13%
3	NRPS			
4	NRPS, NAPAA	stenothricin	NRP: Cyclic depsipeptide	13%
5	T3PKS	herboxidiene	Polyketide	7%
6	ectoine	ectoine	Other	100%
7	NRPS-like			
8	melanin	melanin	Other	60%
9	siderophore	desferrioxamin B / desferrioxamine E	Other	83%
10	butyrolactone			
11	T2PKS	spore pigment	Polyketide	83%
12	lanthipeptide-class-iii	naphthomycin A	Polyketide	9%
13	terpene	julichrome Q3-3 / julichrome Q3-5	Polyketide	25%
14	T1PKS, siderophore	kinamycin	Polyketide	22%
15	thiopeptide, thioamitides, NRPS, ladderane	RP-1776	Polyketide + NRP: Cyclic depsipeptide	10%
16	other, T1PKS	chlorizidine A	NRP + Polyketide: Modular type I	11%
17	NRPS-like	meilingmycin	Polyketide	3%
18	RiPP-like			
19	terpene	geosmin	Terpene	100%
20	siderophore	grincamycin	Polyketide: Type II + Saccharide: Hybrid/tailoring	8%
21	betalactone	belactosin A / belactosin C	Other	75%
22	terpene	hopene	Terpene	92%
23	terpene, T1PKS, NRPS	isorenieratene	Terpene	54%
24	T1PKS, NRPS-like, transAT-PKS-like, lanthipeptide-class-ii	salinosporamide A	Polyketide	23%
25	NRPS, terpene, lanthipeptide-class-i, lanthipeptide-class-ii	cadaside A / cadaside B	NRP	28%
26	NRPS	cyclomarin D	NRP	8%
27	RiPP-like, lanthipeptide-class-iii	informatipeptin	RiPP: Lanthipeptide	57%
28	lanthipeptide-class-iv, PKS-like, lassopeptide	GE81112	NRP	14%

### Identification of aromatic compound degradation genes in *Streptomyces* sp. SCPE-10

The genome of *Streptomyces* sp. SCPE-10 was screened in silico to investigate its potential for degrading aromatic compounds. Annotation revealed a range of genes encoding enzymes classically involved in aromatic hydrocarbon catabolism, including cytochrome P450 monooxygenases, dioxygenases (aromatic ring-hydroxylating and ring-cleaving types), DyP-type peroxidases, and multicopper oxidases. These enzymes mediate essential steps such as hydroxylation, ring cleavage, and phenolic compound oxidation of 2,4-D (Table 4).

Additionally, genes for glutathione S-transferases and epoxide hydrolases suggest a detoxification capability via conjugation and hydrolysis of electrophilic aromatic compounds. KEGG-based pathway analysis indicated genetic potential for the degradation of benzoate, phenylacetate, naphthalene, and chlorinated aromatics. Key enzymes such as protocatechuate 3,4-dioxygenase, catechol 2,3-dioxygenase, and salicylate hydroxylase were consistently annotated. Several of these genes were located within biosynthetic gene clusters (BGCs) predicted by antiSMASH, suggesting a link between secondary metabolism and xenobiotic degradation (Table 5). The combination of these features, highlights the robust genetic capacity of SCPE 10 to metabolize a wide array of persistent aromatic pollutants.

**Table 4:** Main genes identified in the genome of *Streptomyces* sp. SCPE-10 involved in the 2,4-D degradation pathway

Enzymes encoded by the tfd genes of SCPE-10	Contig	KEGG code	Gene	Function
Alpha-ketoglutarate-dependent dioxygenase AlkB	00909	K06912	*tfdA*	Encodes oxygenase’s and dioxygenase’s, which catalyzes the first step in 2,4-D degradation by converting it into 2,4-dichlorophenol (2,4-DCP)
Alpha-ketoglutarate-dependent sulfate ester dioxygenase	02595		*tfdA*	
L-lysine N6-monooxygenase	01973	K10676	*tfdB*	Encodes a phenol monooxygenase, which hydroxylates 2,4-DCP into 3,5-dichlorocatechol, preparing it for ring cleavage
L-lysine N6-monooxygenase	02752		*tfdB*	
Cytochrome P450 monooxygenase PikC	03386		*tfdB*	
Cytochrome P450 monooxygenase PikC	05669		*tfdB*	
Cytochrome P450 monooxygenase PikC	05753		*tfdB*	
Cytochrome P450 monooxygenase PikC	06179		*tfdB*	
Cytochrome P450 monooxygenase PikC	06457		*tfdB*	
putative FAD-linked oxidoreductase	02874		*tfdB*	
putative FAD-linked oxidoreductase YvdP	03794		*tfdB*	
3-phenylpropionate/cinnamic acid dioxygenase ferredoxin–NAD(+) reductase component	01267		*tfdC*	Encodes a catechol 1,2-dioxygenase, which performs ortho-cleavage of the aromatic ring of 3,5-dichlorocatechol
Homogentisate 1,2-dioxygenase	01691		*tfdC*	
Biphenyl 2,3-dioxygenase, ferredoxin component	01879		*tfdC*	
3-carboxy-cis, cis-muconate cycloisomerase	07096		*tfdD*	Encodes a hydrolase, which acts on carboxylated ring-cleavage intermediates to facilitate further breakdown
Alcohol dehydrogenase	00032		*tfdF*	Encodes a dehydrogenase, which oxidizes these intermediates, completing the mineralization of the aromatic compound
NADP-dependent alcohol dehydrogenase C 2	05077		*tfdF*	
Bifunctional cytochrome P450/NADPH–P450 reductase	06636		*tfdF*	
Bifunctional cytochrome P450/NADPH–P450 reductase	06646		*tfdF*	
putative alcohol dehydrogenase AdhA	00735		*tfdF*	
Alcohol dehydrogenase	02702		*tfdF*	
Alcohol dehydrogenase	04748		*tfdF*	
NADP-dependent alcohol dehydrogenase C 2	05077		*tfdF*	
Alcohol dehydrogenase	05463		*tfdF*	
Alcohol dehydrogenase	06744		*tfdF*	
Alcohol dehydrogenase	06941		*tfdF*	

**Table 5:** Analysis of aromatic compound degradation pathways predicted in the genome of *Streptomyces* sp. SCPE-10

Degradation Pathway	KEGG Map Ecs	Genes Detected in SCPE 10	Coverage (%)
Benzoate degradation via hydroxylation	50	12	24.0%
Benzoate degradation via CoA ligation	44	14	31.8%
Naphthalene and anthracene degradation	22	6	27.3%
Toluene and xylene degradation	23	5	21.7%
Chlorobenzoate degradation	13	3	23.1%
Phenol degradation	9	3	33.3%
Catechol ortho- and meta-cleavage	14	4	28.6%
2,4-Dichlorobenzoate degradation	29	3	10.3%
Styrene degradation	8	3	37.5%
Ethylbenzene degradation	11	4	36.4%
Dioxin degradation	12	3	25.0%
1,2-Methylnaphthalene degradation	17	7	41.2%
Cyclohexanol degradation (Baeyer - Villiger)	10	2	20.0%
Metabolism of xenobiotics by cytochrome P450	7	3	42.9%
Fluorobenzoate degradation	10	1	10.0%

## Discussion

Aromatic compounds and herbicide derivatives such as 2,4-D are among the most persistent and hazardous pollutants in agricultural soils, posing significant ecological and health risks [1, 11]. Microbial biodegradation stands out as an efficient, cost-effective, and environmentally friendly approach for remediating contaminated environments [12]. Several bacterial genera, including *Pseudomonas*, *Bacillus*, *Rhodococcus*, *Sphingomonas*, and *Streptomyces*, have been explored for their biodegradation capabilities [13–16]. Among them, *Streptomyces* spp. are notable for their genetic diversity and enzymatic arsenal that allow them to degrade a wide range of complex xenobiotics [17]. In this study, we isolated and characterized the strain *Streptomyces* SCPE-10, which was able to grow in the presence of 2,4-D, highlighting its resilience to toxicity and adaptation to polluted environments. Genomic analysis revealed several genes associated with hydrocarbon and aromatic compound catabolism, including cytochrome P450 monooxygenases, ring-cleaving dioxygenases, hydrolases, transferases, and dehydrogenases, enzymes already known for their role in aromatic degradation pathways [18, 19]. KEGG-based annotation identified several pathways related to the degradation of benzoate, naphthalene, toluene, ethylbenzene, fluorene, and other persistent aromatics. Genes such as *tfd*, *pcaG*, *catA*, *xylE*, and *ligB* were found to participate in aromatic ring cleavage via catechol and protocatechuate intermediates, leading to the production of tricarboxylic acid cycle intermediates [20–22]. AntiSmash analysis revealed 28 biosynthetic gene clusters, many associated with aromatic structures or detoxification mechanisms. These included *terpenes*, *siderophores*, *ectoines*, *lanthipeptides*, and complex *NRPS/PKS* clusters, underscoring the ecological versatility of SCPE 10 and its potential for biotechnological exploitation, especially in producing novel metabolites [23–25]. Notably, genes such as *adhP*, *aldH*, *fadB*, and *paaF* involved in the degradation of alkenes and halogenated aromatics were also identified, supporting SCPE 10’s capability to degrade 2,4-D and related compounds. Additionally, the presence of *ubiX*, associated with anaerobic aromatic degradation, suggests that the strain may maintain metabolic plasticity under variable environmental conditions [26, 27].

Overall, *Streptomyces* sp. SCPE-10 is a promising candidate with potential for the bioremediation of contaminated soils and may serve as a platform for the development of microbial consortia targeting xenobiotic degradation in agroecosystems. Further studies should focus on the identification of end-products, transcriptomic profiling under environmental stress, and validation of degradation efficiency in mesocosm or field-scale trials.

## Conclusion

*Streptomyces* sp. SCPE-10 exhibited a remarkable genomic capacity to degrade aromatic compounds, highlighting its biotechnological potential for environmental remediation. Genomic analysis revealed a diverse set of catabolic genes and biosynthetic clusters linked to xenobiotic metabolism, particularly enzymes such as cytochrome P450s, aromatic ring-cleaving dioxygenases, hydrolases, and multiple NRPS/PKS clusters. These findings position SCPE-10 as a promising microbial resource for bioremediation strategies targeting herbicide-contaminated environments.

## Data Availability

This genome sequencing project of *Streptomyces* sp. SCPE-10 has been deposited in the NCBI genome database under NCBI Reference Sequence: SUB15461668. The BioProject and BioSample accessions are PRJNA1291388 and SAMN49962064, respectively.
